# The impact of large language models on radiology: a guide for radiologists on the latest innovations in AI

**DOI:** 10.1007/s11604-024-01552-0

**Published:** 2024-03-29

**Authors:** Takeshi Nakaura, Rintaro Ito, Daiju Ueda, Taiki Nozaki, Yasutaka Fushimi, Yusuke Matsui, Masahiro Yanagawa, Akira Yamada, Takahiro Tsuboyama, Noriyuki Fujima, Fuminari Tatsugami, Kenji Hirata, Shohei Fujita, Koji Kamagata, Tomoyuki Fujioka, Mariko Kawamura, Shinji Naganawa

**Affiliations:** 1 Department of Central Radiology, Kumamoto University Hospital, Honjo 1-1-1, Kumamoto, 860-8556 Japan; 2 Department of Radiology, Nagoya University Graduate School of Medicine, Nagoya, Aichi Japan; 3 Department of Diagnostic and Interventional Radiology, Graduate School of Medicine, Osaka Metropolitan University, 1‑4‑3 Asahi‑Machi, Abeno‑ku, Osaka, 545‑8585 Japan; 4 Department of Radiology, Keio University School of Medicine, Shinjuku‑ku, Tokyo, Japan; 5 Department of Diagnostic Imaging and Nuclear Medicine, Kyoto University Graduate School of Medicine, Sakyoku, Kyoto Japan; 6 Department of Radiology, Faculty of Medicine, Dentistry and Pharmaceutical Sciences, Okayama University, Kita‑ku, Okayama, Japan; 7 Department of Radiology, Osaka University Graduate School of Medicine, Suita City, Osaka, Japan; 8 Department of Radiology, Shinshu University School of Medicine, Matsumoto, Nagano, Japan; 9 Department of Diagnostic and Interventional Radiology, Hokkaido University Hospital, Sapporo, Japan; 10 Department of Diagnostic Radiology, Hiroshima University, Minami‑ku, Hiroshima, Japan; 11 Department of Diagnostic Imaging, Graduate School of Medicine, Hokkaido University, Kita‑ku, Sapporo, Hokkaido Japan; 12 Department of Radiology, University of Tokyo, Bunkyo‑ku, Tokyo, Japan; 13 Department of Radiology, Juntendo University Graduate School of Medicine, Bunkyo‑ku, Tokyo, Japan; 14 Department of Diagnostic Radiology, Tokyo Medical and Dental University, Bunkyo‑ku, Tokyo, Japan

**Keywords:** Diagnostic radiology, Artificial intelligence, Deep learning, Large language model, Radiological workflow

## Abstract

The advent of Deep Learning (DL) has significantly propelled the field of diagnostic radiology forward by enhancing image analysis and interpretation. The introduction of the Transformer architecture, followed by the development of Large Language Models (LLMs), has further revolutionized this domain. LLMs now possess the potential to automate and refine the radiology workflow, extending from report generation to assistance in diagnostics and patient care. The integration of multimodal technology with LLMs could potentially leapfrog these applications to unprecedented levels.

However, LLMs come with unresolved challenges such as information hallucinations and biases, which can affect clinical reliability. Despite these issues, the legislative and guideline frameworks have yet to catch up with technological advancements. Radiologists must acquire a thorough understanding of these technologies to leverage LLMs’ potential to the fullest while maintaining medical safety and ethics. This review aims to aid in that endeavor.

## Introduction

The inception of Deep Learning (DL) has catalyzed a significant progression in artificial intelligence (AI) [1], unlocking numerous possibilities, especially in diagnostic radiology—an arena pivotal for accurate imaging data interpretation. This progression is attributed mainly to the emergence of Convolutional Neural Networks (CNNs) [2, 3], which have markedly enhanced image recognition, segmentation, analysis, and improvement of image quality [1, 4–15]. This represents a foundational shift in automated feature extraction from imaging data, consequently reducing the time and expertise required for interpreting medical images. Additionally, DL-powered tools have demonstrated their efficacy in improving diagnostic accuracy by aiding radiologists in precisely detecting anomalies such as tumors, external injuries, and other pathological conditions [16–20]. These advancements not only accelerate the diagnostic process but also contribute substantially to prognostic evaluations, thus playing a crucial role in elevating patient care and outcomes [21].

The introduction of the Transformer architecture has been a significant milestone in machine learning, paving the way for the development of Large Language Models (LLMs) such as the Generative Pre-trained Transformer (GPT) series. The architecture’s proficiency in handling sequential data efficiently through attention mechanisms has expedited the evolution of LLMs, which now possess the ability to understand and generate human-like text with remarkable accuracy. The subsequent advent of ChatGPT further accentuated the popularity and utility of LLMs by showcasing their capability to engage in more natural, dynamic dialogues, thus expanding the scope of applications across various fields. In diagnostic radiology, LLMs might offer a promising pathway for enhancing multiple aspects of the radiology workflow. Their capability to automate report generation and expedite information retrieval can potentially save significant time for radiologists, thereby ameliorating the efficiency and accuracy of diagnostic processes.

Despite the undeniable utility of LLMs, there has been a scarcity of reviews describing the rapid development of LLMs for clinical radiologists. This article delineates a brief history of contemporary LLMs and provides a synopsis of their application in radiology for the clinical radiologist.

## Overview of DL and LLM before transformer architecture

**Fig. 1:**
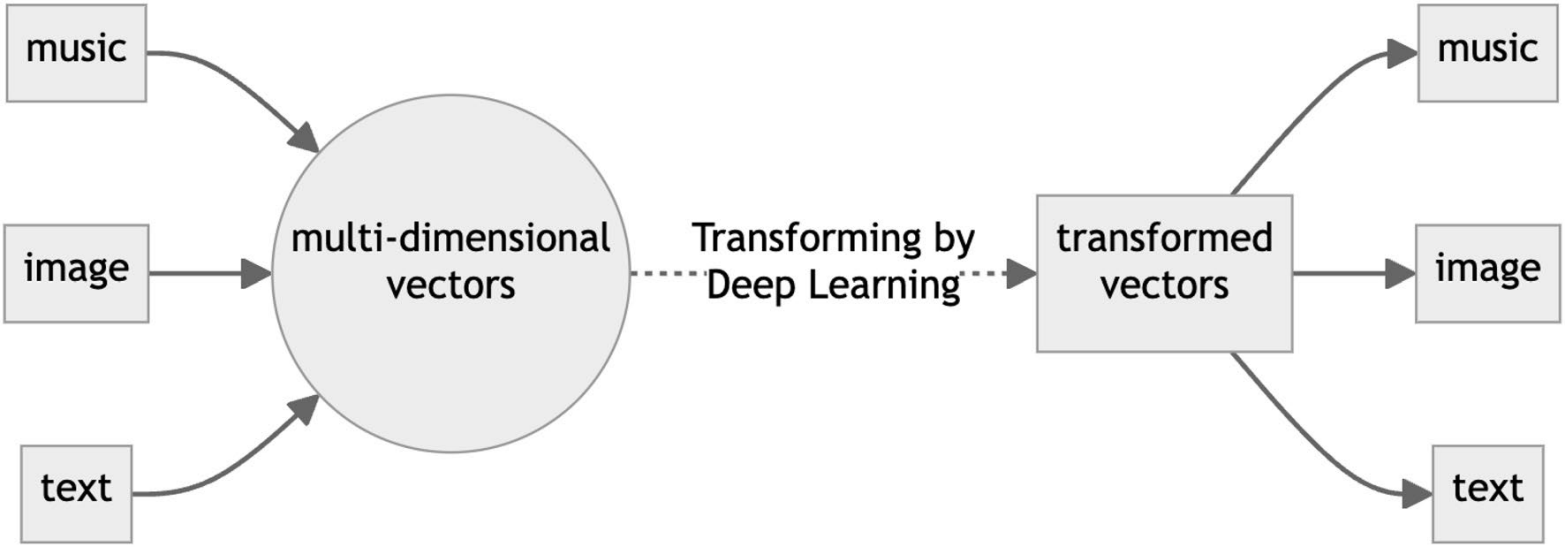
Overview of the Deep Learning Process. If there is some relationship between the matrices representing input and output data, Deep Learning can learn it given a myriad of training data by the “universal approximation theorem”

Natural Language Processing (NLP), CNN-based image processing, is a branch of AI. Recently, DL has been employed extensively in NLP tasks. This wide applicability of DL can be attributed to the universal approximation theorem [22]. This theorem suggests that a neural network, provided with enough layers and neurons, can approximate any reasonable function with a high degree of accuracy. DL thus operates by approximating an ideal function capable of transforming various data types, such as images, music, and text, into other forms of data (Fig. 1). In broad terms, the current LLM process involves generating a response sentence from a given request sentence, essentially transforming multi-dimensional vectors representing the request sentence into multi-dimensional vectors representing the response sentence. Despite the development of various DL models for language processing applications, this fundamental concept remains constant.

Before the inception of the Transformer architecture, the domain of NLP chiefly relied on architectural frameworks such as Recurrent Neural Networks (RNNs) [2], Long Short-Term Memory networks (LSTMs) [23], and CNNs. RNNs, with their intrinsic capability to encapsulate sequential information, were predominantly employed for an array of NLP tasks including but not limited to translation, sentiment analysis, and named entity recognition. However, they frequently encountered challenges with long-term dependencies owing to the vanishing or exploding gradient dilemma. To alleviate these issues, LSTMs were introduced as a special kind of RNN capable of learning long-term dependencies, providing a more robust framework for handling sequences and time-series data. Nonetheless, while LSTMs mitigated the gradient problem to an extent, they still entailed a significant computational and temporal demand, especially as data complexity increased. Conversely, CNNs were more adept at local pattern recognition within data and found their application in certain NLP tasks, yet they too were encumbered by limitations in capturing long-range dependencies within text sequences. Moreover, the computational and temporal demands of training these networks escalated significantly, especially in the face of the burgeoning size and intricacy of data.

**Fig. 2:**
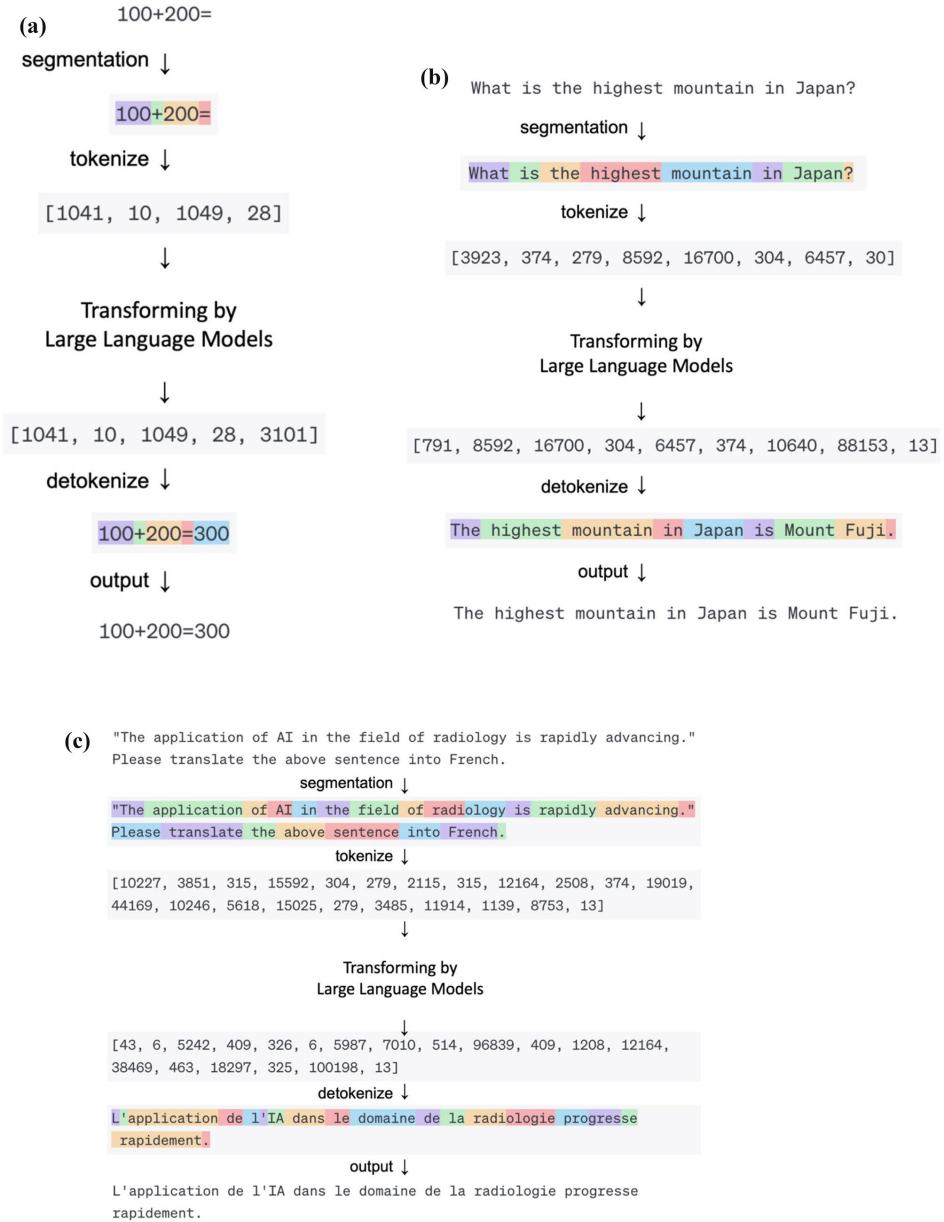
Various Language Processing with Large Language Model. Examples of **a** computation, **b** conversation, and **c** translation, respectively. All of these different language processing tasks can be accomplished using the same process: converting input data into a matrix using a tokenizer, transforming it into another matrix using a Large Language Model, and then converting it back into output data

Recently, it has been elucidated that the efficacy of modified RNN can be commensurate with that of newer models [24], contingent upon the scale. However, the formidable computational costs associated with their operation pose a significant deterrent, leading to their diminished utilization in recent times. It is also recognized that the architectural distinctions among these models exert a lesser impact on performance compared to the magnitude of parameters encompassed [25]. Hence, the details of the structure of these models are omitted from this review. The cornerstone of operation for these NLP frameworks, inclusive of the subsequent Transformer architecture delineated, hinges on the initial transmutation of textual sentences into a numerical sequence termed as tokens, facilitated by a tokenizer. Figure 2 shows an example of sentence conversion by an online Tokenizer (https://platform.openai.com/tokenizer). This token sequence subsequently undergoes a further transmutation into an alternate numerical sequence. This tokenization process has been used in NLP even before the rapid development of Deep Learning, and the basic principle is the same in recent LLM.

## Advent and evolution of the transformer architecture for LLM

**Fig. 3:**
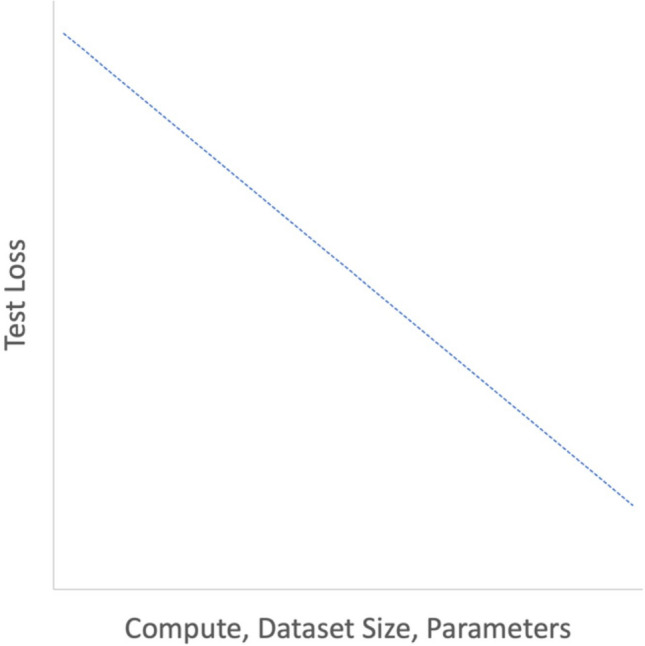
Schema of Scaling Law. The performance of the Transformer follows a simple power law, where the parameters, dataset size and computational resources are considered as variables. For instance, if the other two variables are not the bottleneck, doubling the number of parameters results in a performance improvement by a power of 2. (Graphs are plotted on logarithmic scales)

The advent of the Transformer architecture marked a significant milestone in the realms of NLP and machine learning [26–28]. Unlike previous architectures, the Transformer was designed to efficiently handle parallel processing, making it especially suitable for training on graphics processing units. This novel design facilitated a substantial reduction in training times and effective management of large datasets, enabling the training of large-scale models that were previously unfeasible. Furthermore, this escalation in learning scale elucidated relationships known as scaling laws (Fig. 3), which delineate the relationships between model size, dataset size, and the amount of computing used for training [25]. This study reported the performance of language models on the cross-entropy loss scales according to a power-law to these factors.

The scalability and parallel processing capabilities of the Transformer architecture accelerated the development of large-scale neural network models. Notably, the Generative Pre-trained Transformer (GPT) [25, 29, 30] and Bidirectional Encoder Representations from Transformers (BERT) [31] stand as exemplary embodiments of the large-scale expansion and advancement of the Transformer. GPT, developed by OpenAI, is an LLM based on the Transformer architecture, focusing on predicting the next word in a given text sequence. It is generally pre-trained on a vast text corpus and then fine-tuned for specific tasks. The GPT series (GPT-1, 2, 3, 3.5, and 4) aims to enhance performance by augmenting model size, with GPT-3 boasting 175 billion parameters [25]. On the other hand, BERT, developed by Google, also leverages the Transformer architecture but adopts a different approach. By considering bidirectional context, BERT achieves superior performance on specific NLP tasks, which is particularly advantageous in tasks like question-answering and named entity recognition.

In any case, as the scale increases, the performance of language models on tasks has significantly improved. Particularly, large-scale models like GPT-3 have been reported to exhibit excellent performance on entirely new tasks without retraining or with just a few demonstrations [25]. This burgeoning performance with scale underscores the remarkable potential and evolution propelled by the Transformer architecture, contributing to the broad spectrum of advancements in NLP and machine learning fields.

## LLM limitations

Despite the remarkable achievements, LLMs have inherent limitations. One of the notable issues is hallucination, where the model generates incorrect or fictional information that wasn’t present in the training data [32–36]. This problem arises due to several underlying factors and poses challenges to the implementation and trustworthiness of LLMs, especially in critical fields like healthcare. A notable cause of hallucination, the source-reference divergence, arises from heuristic data collection methods or inherent challenges in certain natural language generation tasks, leading to deviations from the provided source during text generation. Similarly, exploitation through 'jailbreak' prompts that were not intended by the developers, which manipulate the model’s behavior or output, and reliance on datasets with incomplete or contradictory information significantly influence the LLM’s generated responses. These issues are exacerbated by misleading training data, where incorrect, outdated, or biased information is propagated into the generated outputs, further undermining the reliability of LLMs in a clinical setting. Mitigation strategies aimed at reducing hallucination in LLMs include the employment of regularization techniques, augmenting training data, and leveraging few-shot learning strategies. However, completely preventing hallucination remains a formidable challenge due to the inherent limitations of the current LLM architectures and the vast and varied nature of the training data.

Inductive biases [37] refer to the set of assumptions that a model makes to predict outputs for unseen data. In LLMs, these biases might arise from the training data, leading the model to generate outputs that may not align with real-world scenarios. The performance and the model’s capacity to generalize across varying contexts can be adversely affected by these biases. Additionally, the “black box” nature of LLMs denotes the lack of transparency in understanding how the model arrives at a particular decision, which is a critical requirement for real-world applications, particularly in fields demanding explainability like medical fields.

The output generated by LLM can be inaccurate and misleading due to these limitations and can lead to misguided clinical problems [38], and LLM output should be carefully evaluated by professionals.

## Release of ChatGPT and its application to medical fields

The public release of ChatGPT on November 30, 2022, developed by Open AI, heralded a new era of accessibility, drawing a plethora of users from diverse fields. The transition to GPT-3.5 used in ChatGPT was a pivotal moment, as the incorporation of Reinforcement Learning from Human Feedback (RLHF) [39] played a crucial role in refining the model's responses, making them more coherent and contextually appropriate. This widespread adoption triggered a boom, as the model's potential in various applications was explored extensively. Additionally, GPT-4 is slated for public availability on March 14, 2023. While the specific architectural details remain undisclosed, it is anticipated that GPT-4 will herald enhanced performance across diverse domains, marking a substantial advancement from its predecessor, GPT-3.5.

In the medical field, ChatGPT showcased an impressive aptitude by excelling in medical examinations [40], a testament to its proficiency in handling medical knowledge. Furthermore, studies have highlighted its competence in real-world medication consultations [41], where it displayed a higher appropriateness rate in responding to public consultation questions compared to those posed by healthcare providers in a hospital setting. Although ChatGPT’s official warnings mention its use in diagnostics, saying, “Making automated decisions in domains that affect an individual’s rights or well-being (e.g., law enforcement, migration, management of critical infrastructure, safety components of products, essential services, credit, employment, housing, education, social scoring, or insurance)” (https://openai.com/policies/usage-policies) and the use of ChatGPT on WWW may have a serious concern of data leaking unless user manifest opt-out (https://privacy.openai.com/policies), these achievements highlight ChatGPT’s potential in giving important medical insights.

## Capable applications of LLM in the radiology field

Radiologists routinely engage with a substantial volume of textual information encompassing diagnostic request forms, medical charts, reports from other examinations, references to various guidelines and past literature, diagnostic imaging reports, and the generation of scholarly articles. However, recent years have witnessed an uptrend in the utilization of imaging diagnostic modalities across numerous countries. The ensuing amplification in image interpretation and reporting duties has precipitated concerns surrounding burnout among radiologists [42]. Despite the aforementioned limitations, LLMs hold promise as potential adjunctive tools to ameliorate the burden associated with such radiological endeavors.

The accelerated development and refinement of LLMs such as ChatGPT have catalyzed a notable performance in medical examinations. For instance, an evaluation of ChatGPT on the United States Medical Licensing Exam (USMLE) revealed that it performed at or near the passing threshold across all three exams without any specialized training or reinforcement [40]. Moreover, in a radiology board-style examination, ChatGPT nearly met the passing criteria without specific radiology pre-training, while a GPT-4 demonstrated superior performance compared to GPT-3.5, indicating a significant advancement in model capability [43]. ChatGPT based on GPT-4 scored 65% when answering Japanese questions from the Japan Radiology Board Examination (JRBE) [44]. Another study evaluated ChatGPT's performance on the Polish specialty exam in radiology and diagnostic imaging. Although ChatGPT did not reach the passing threshold of 52%, it came close in certain question categories [45]. Although the precise performance of LLMs may exhibit variance based on language [46], these facts suggest that LLMs may be able to play a supplementary role even in quite specialized radiology work, even if only for text data at this point.

Given the demonstrated capabilities of LLMs, there exists a potential to significantly enhance radiological workflow. This enhancement may manifest through proficient summarization of medical records, streamlined diagnostic imaging studies, clinical decision-making, rewriting, and generation of radiology reports [47, 48]. For instance, while not a general-purpose LLM akin to GPT-4, an LLM trained specifically on medical data, known as PubMedBERT [49], has been reported to accurately predict mortality within 24 h of admission for patients in intensive care units using solely medical record data [50]. This demonstrates the capacity of LLMs to adeptly handle and derive meaningful insights from textual data such as medical records, extending promise for their application in critical care settings. Additionally, There is a preliminary study that helps to automatically determine imaging studies and protocols based on the radiology department's request form [51]. Another paper demonstrates that the DL-based NLP model can accurately classify the status of bone metastasis in Japanese radiology reports, providing a potential tool for the early and efficient detection of patients with bone metastasis. [52] Given such capabilities, it is conceivable that soon, LLMs could semi-automatically configure protocols for imaging examinations such as CT or MRI, based on the information extracted from examination request forms, medical chart data, and other diagnostic data.

Furthermore, there are several reports of rewriting radiology reports written by radiologists using LLM. The utility of structured reporting in radiology has been acknowledged, yet it has also been reported that LLMs can rewrite free-style reports into structured reports [53]. This not only holds promise for daily clinical practice but also for the education of radiology residents. Additionally, while the interpretation of radiology reports necessitates specialized knowledge, it has been reported that LLMs are capable of translating these specialized reports into more comprehensible language for a general patient [54]. This potential application anticipates aiding in patient communication and comprehension, further extending the scope of LLMs in enhancing patient-centered care in radiology.

## Report generation assistance through LLMs

One of the most direct applications of LLMs in reducing workload within the clinical setting could arguably be in assisting with the generation of imaging diagnostic reports themselves. While there have been numerous reports on this subject from earlier times [55–57], the advent of the GPT series has marked a notable advancement. It has been reported that LLMs can autonomously generate human-like radiology reports from merely brief keywords, and the differential diagnoses provided are relatively reliable [58]. This suggests a significant potential for augmenting the efficiency and accuracy of report generation, a critical component of the radiological workflow.

The approach delineated in this paper [58] for aiding the generation of radiology reports hinges solely on the utilization of ChatGPT, obviating the need for any specialized training and hence, rendering it a reproducible methodology accessible to all. The requisite inputs for this process are limited to basic demographic data such as age and gender, keywords embodying the imaging findings, and a prompt tailored for report generation. However, it is noteworthy that the prompt necessitates customization for imaging report generation; the prompt utilized in this paper follows a structured format aimed at aiding the generation of radiology reports using ChatGPT. This structure encompasses three pivotal components: (1) establishing the role of the LLM as a radiology specialist, (2) specifying the output sections (Findings, Impression, Differential Diagnosis), and (3) elaborating on the content for each of these sections. This structured approach is predicated on previously reported guidelines for radiology reporting [59], thereby adhering to the established norms within the radiological community.

**Fig. 4:**
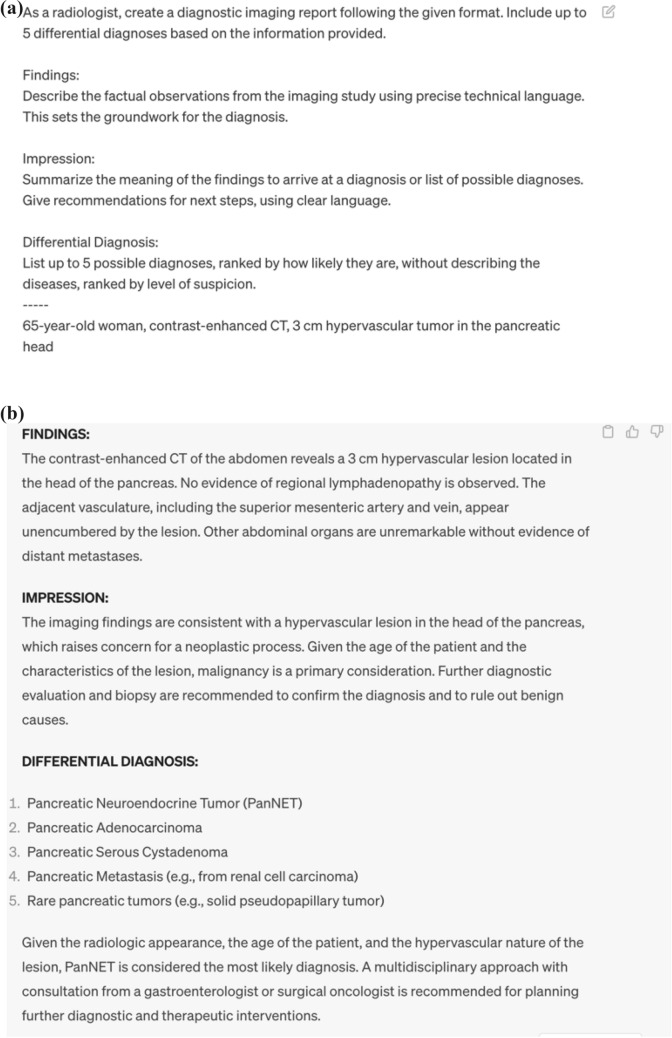
Example of Radiology Report Generated by GPT-4. **a** Prompts and **b** Corresponding generated reports. Providing specific instructions on the desired role, format, and content of output items within the prompts can enhance the quality of GPT-4 output

Figure 4 shows the simplified prompt for generating radiology reports and usage examples. The prompt used in this review is as follows: “As a radiologist, create a radiology report following the given format. Include up to 5 differential diagnoses based on the information provided. Findings: Describe the factual observations from the imaging study using precise technical language. This sets the groundwork for the diagnosis. Impression: Summarize the meaning of the findings to arrive at a diagnosis or list of possible diagnoses. Give recommendations for the next steps, using clear language. Differential Diagnosis: List up to 5 possible diagnoses without describing the diseases, ranked by level of suspicion.” By typing simple keywords followed by a prompt like this on OpenAI's ChatGPT site (https://chat.openai.com/), anyone can generate something like a radiology report without any additional training.

However, there are inherent challenges that must be addressed to ensure the safe and effective deployment of LLMs in this context. One such challenge is the phenomenon of hallucination, where the model generates incorrect or misleading information. This aspect necessitates a cautious approach to employing LLMs for diagnostic reporting. The potential for hallucinations to misguide clinical interpretations underscores the importance of having appropriate regulatory frameworks in place to mitigate risks associated with the use of LLMs in clinical settings.

Moreover, the legal and ethical frameworks surrounding the application of LLMs for diagnostic reporting need to be robustly established. As described above, today, even those without a background in diagnostic radiology can easily generate a large number of reports that are difficult to distinguish from diagnostic reports using LLMs. This has significant implications for the medical field. Given the potential for misinterpretation or misuse of these reports, it is crucial that regulations are put in place to ensure that only qualified professionals are authorized to interpret and apply these findings. Ensuring the responsible use of LLMs while maximizing their potential to reduce the workload and improve the accuracy and efficiency of diagnostic reporting requires a balanced approach. The evolution of legal and professional guidelines, in tandem with technological advancements, is imperative to foster a conducive environment for the integration of LLMs in radiological practice, ensuring both patient safety and enhanced clinical workflow.

## Potential of LLMs in research work

The advent of LLMs like ChatGPT might also bring forth a promising avenue for alleviating the burgeoning workload in radiological research. It has been reported that LLM’s text generation capability has reached a level close to that of humans in the research field [60]. In this study, researchers asked a chatbot to generate 50 medical research abstracts based on excerpts published in JAMA, The New England Journal of Medicine, BMJ, The Lancet, and Nature Medicine. They then compared these generated abstracts with the original ones and asked a group of medical researchers to identify any fabricated abstracts. Scientists fared a correct identification rate of 68% for generated abstracts and 86% for genuine ones; however, they also made mistakes, incorrectly classifying 32% of the generated abstracts as genuine and 14% of the genuine abstracts as generated. An emblematic instance is that a pre-peer review version of the paper evaluating ChatGPT's performance at USMLE added ChatGPT to the authors [40]. Furthermore, LLMs can serve as invaluable adjuncts in review processes, assisting researchers and reviewers in tasks such as text summarization, extraction, and past literature retrieval. This assistance could be particularly beneficial for non-native authors, facilitating a smoother and more coherent review process [61].

On the contrary, a very recent pre-peer review paper examines the possibility of replacing the entire peer review process with LLM [62]. In this study, authors compared the feedback generated by GPT-4 and human peer reviewers, it was found that the overlap rate of points identified by GPT-4 and human reviewers was 30.85% on average in the Nature journal and 39.23% in the International Conference on Learning Representations (ICLR) conference. These rates were comparable to the overlap rate between two human reviewers, which averaged 28.58% in the Nature journal and 35.25% in the ICLR conference. Overall, 57.4% of users evaluated the feedback from GPT-4 as useful or very useful, and 82.4% felt that the feedback from GPT-4 was more beneficial than at least some of the feedback from human reviewers.

However, the integration of LLMs into the scholarly landscape is not devoid of ethical and procedural considerations. One primary concern pertains to authorship, as LLMs, lacking the capacity for responsibility, cannot be listed as authors despite their substantial contribution to manuscript creation [63, 64]. Most academic papers’ submission guidelines have acknowledged this concern by mandating a detailed disclosure if LLMs are employed in the manuscript preparation, ensuring a transparent acknowledgment of LLM assistance. Moreover, a cautious approach towards the utilization of LLMs in review processes is advocated to mitigate risks associated with confidentiality and other potential malfeasances. While there has been a paucity of guidelines on the prudent use of LLMs in reviews, the recently published guidelines by Radiology [65] underline the importance of cautious employment, with a particular emphasis on maintaining confidentiality. This prudent approach towards LLM utilization not only fortifies the integrity of the review process but also sets a precedent for fostering responsible AI integration in radiological research.

## Future outlook of LLMs in radiology

LLM is currently evolving rapidly, and multimodal technology seems to be one of the most notable and relevant in the field of radiology. Like LLM, this technology is based on transformer architecture, but it can also handle image data in a unified manner. Microsoft's Bing AI is currently compatible with this multimodal technology and is also available to paid users of Open AI’s GPT-4. Currently, the main reports revolve around annotations of images and videos. However, there is also mention of the potential of AI trained on medical data [66]. The integration of multimodal technology into LLM, or AI in medical imaging, might bring a new dimension to radiology. According to prior research, the integration of multimodal technology has the potential to revolutionize the precision of image diagnosis in the field of diagnostic radiology [67]. Moreover, its implementation could substantially reduce the time required for image analysis. However, given that, as with research work, LLMs are not responsible and may produce reports that seem authoritative with a completely different meaning through hallucinations, etc., and given that multimodal technology is prepared to be used by people with no knowledge of the radiology field, it Given that multimodal technology may prepare people with no knowledge of radiology to use it, legal development and guidelines might be needed for the application of this technology in radiology.

Another very promising outcome is the mitigation of LLM hallucinations. One way to overcome hallucinations in LLMs is by improving the training data. The quality and diversity of the training data play a significant role in the performance of these models. There have been reports on LLMs specific to the medical side, and if these models are developed, hallucination could be significantly reduced. Another method is to combine with search. The integration of search into LLMs can help in reducing the frequency of hallucinations by recognizing and rectifying incorrect or nonsensical generated text. The third approach is to refine the model architecture and learning methods of the neural network. Recent literature has reported the potential for significantly improved performance over existing LLMs by combining conventional neural network methods with meta-learning for compositionality [68]. For instance, it has the potential to operate efficiently even when faced with unfamiliar words or concepts, thereby substantially minimizing the requisite volume of learning data. It is anticipated that such advancements will persistently emerge in the future.

As the development of LLMs is expected to advance further, it is also anticipated that the potential risks associated with this will increase. As mentioned earlier, examples such as radiology report generation, scientific review, and medical consultation on social media, there is a possibility that LLMs will be used not as copilots, but as almost independent agents for some purposes, and in extreme cases, it cannot be denied that even those who have no knowledge of radiology or medicine may provide services. However, there is no method to completely solve the problems of LLMs such as hallucinations and biases at present. In addition, LLMs implicitly memorize the information contained in the training data, and there is a possibility that personal information or medical information may be included in the generated text. Even if LLMs develop, their output may be inaccurate or inappropriate and may affect the health and safety of patients. Developers and users of LLMs in radiology work should use them with a correct understanding of their capabilities and limitations, and checking the output of LLMs by radiologists will continue to be essential in the future.

LLMs have the potential to bring innovation to the medical field, but on the other hand, they also have the potential to bring crisis to the medical field. The development and application of LLMs to the medical field should be done carefully and responsibly, but at present, the rapid development of technology has not caught up with the establishment of guidelines and laws for the use of LLMs in radiology work. As guidelines and submission rules have been changed for the paper submission and the scientific review, similar preparations are urgently needed for daily radiology work considering the future development of LLMs.

## Conclusion

As LLM continues to mature and evolve, its incorporation into diagnostic radiology harbors immense potential for advancing this field. However, the speed at which technology has developed has outpaced the establishment of consensus, guidelines, and legislation for LLM use. Currently, LLM models serve a “copilot” role, but shortly, they will gain the ability to function as an autonomous “agent”. as demonstrated by tasks such as report generation and paper review mentioned earlier. Nevertheless, this advancement encompasses an array of potential pitfalls concerning medical safety and ethics. A thorough understanding of LLM by radiologists and collaboration with experts is crucial for successfully integrating LLM into radiology.

## Abbreviations

AI: Artificial intelligence
BERT: Bidirectional encoder representations from transformers
CNN: Convolutional neural networks
CT: Computed tomography
DL: Deep learning
GPT: Generative pre-trained transformer
ICLR: International conference on learning representations
JRBE: Japan radiology board examination
LLM: Large language models
LSTM: Long short-term memory
MRI: Magnetic resonance imaging
NLP: Natural language processing
RNN: Recurrent neural networks
RLHF: Reinforcement learning from human feedback
RSNA: Radiological society of North America
USMLE: United States medical licensing examination
